# Prevalence of STIs, sexual practices and substance use among 2083 sexually active unmarried women in Lebanon

**DOI:** 10.1038/s41598-021-89258-5

**Published:** 2021-05-10

**Authors:** Sara Abu Zaki, Jihane Naous, Antoine Ghanem, Diana Abou Abbas, Roland Tomb, Jade Ghosn, Ayman Assi

**Affiliations:** 1 Marsa Sexual Health Center, Beirut, Lebanon; 2 Department of Family Medicine, American University of Beirut Medical Center, Beirut, Lebanon; 3 Faculty of Medicine, University of Saint-Joseph, Beirut, Lebanon; 4 Service des Maladies Infectieuses et Tropicales, AP-HP Nord, Hôpital Bichat - Claude Bernard, Paris, France; 5 INSERM IAME - UMR 1137, Université de Paris, Paris, France; 6 Faculté de Médecine Site Paris Nord, Université de Paris, Paris, France

**Keywords:** Hepatitis, HIV infections, Epidemiology

## Abstract

Access to sexual and reproductive health in conservative communities and in the MENA region are particularly limited and, as such, increase women’s vulnerability to unwanted pregnancies, unsafe abortions, and sexually transmitted infections (STIs). The aim was to assess the prevalence of STIs, sexual practices, recreational drug-use and their possible associations among cisgender unmarried women residing in Lebanon. Data on demographics, sexual practices and substance-use were collected from 2083 unmarried cisgender women who voluntarily attended a sexual health clinic in Lebanon between 2015 and 2019. They tested for HIV, Hepatitis B, Hepatitis C and Syphilis through rapid testing. Other infections (genital warts, Neisseria gonorrhea/Chlamydia trachomatis) were screened for. Regression models were computed between variables. There were two cases of HIV, one of Hepatitis B and syphilis, and no cases of Hepatitis C. Genital warts were present in 15% and symptoms indicative of Neisseria gonorrhea/Chlamydia trachomatis in 14%. Inconsistent condom-use (81%) was significantly associated with number of partners (adj. OR: 0.4). Inconsistent condom-use discussion with partners (33%) was significantly associated with unemployment (adj OR: 1.7), recreational drug-use (adj. OR: 1.4), and number of partners (adj. ORs 3.7–4.4). Unwanted pregnancies (11%) were significantly associated with age (adj. ORs 0.1–0.37), recreational drug-use (adj. OR: 2), using intrauterine device (adj. OR:2.9) and natural birth control methods (adj. OR: 2.4). Recreational drug-use (33%) was significantly associated with age (adj ORs 1.9–2.2), and smoking status (adj. OR: 0.6). The results indicate an urgent need for: (1) Accessible, non-stigmatizing, and inclusive sexual health services dedicated to women’s sexual health; (2) Comprehensive and non-stigmatizing sexual health education for all, but especially women, in order to promote safer sexual practices and effective decision making with regards to contraception and condom-use.

## Introduction

Sexual and reproductive health services (SRH) are instrumental for the health and well-being of individuals and access to SRH is a vital aspect of their human rights. For women, limited access to SRH has been shown to have direct consequences on their mortality and life expectancy. Indeed, sexual and reproductive health disorders are considered among the leading causes of women’s morbidity worldwide^1^. Even though the correlation between limited access to SRH and negative health outcomes for women is quite obvious, sexual and reproductive health and rights of women remain restricted in many parts of the world^2^.

In conservative communities, access to SRH and sexual health education are particularly limited and, as such, increase women’s vulnerability to unwanted pregnancies, unsafe abortions, and STIs^3^. Even in areas where condoms are easily available, many women may refrain from discussing condom-use with their sexual partners due to stigma and reliance on male partners for providing condoms^4^. Moreover, substance-use is still stigmatized in many parts of the world and has been linked to risky behavior including risky sexual practices such as inconsistent condom-use^5–7^. For sexually active women who use substances, the double stigma can increase the likelihood of engaging in risky practices and can also further limit their access to SRH services^8^.

Different social movements across the world, led by women and other marginalized groups, have been striving to claim their sexual rights and autonomy for decades. Gender and sexuality are still part of the continuous political and social struggles in the Middle East and North Africa (MENA) region specifically. Restrictions on women’s sexual autonomy continue in the region, largely, due to social and religious taboos as well as gender inequalities^9^. As such, the MENA region still faces challenges in securing the sexual and reproductive health and rights of women such as shortage of skilled professionals, limited availability of quality SRH, and the de-prioritization of the sexual health needs of women^10^. In MENA, unmarried women particularly struggle to access sexual health information and services as extramarital sex, criminalized in some countries, is still highly stigmatized and can lead to violence or “honor” killing.

Furthermore, the World Health Organization reported a global increase in STI prevalence in 2019^11^. If STIs are left untreated they can significantly deter the health of individuals. For women, late detection can lead to pelvic inflammatory disease (PID), complications during pregnancy and/or labor, or sterility. Among STIs, HPV could lead to cervical cancer, the 3rd most common cancer among female born individuals globally^12^.

Even with the various challenges that compromise the sexual and reproductive health of women worldwide, scientific literature on women’s sexual health remains scarce globally, especially in Lebanon and the MENA region. The lack of sufficient literature on the topic deters the generation of context-relevant evidence-based strategies to overcome challenges and improve the sexual health of women.

Therefore, this study aims to assess the prevalence of STIs as well as sexual practices, recreational drug-use and their possible associations among cisgender unmarried women.

## Methods

### Sample

Data were collected from beneficiaries who had voluntarily accessed a sexual health center in Beirut, Lebanon between January 2015 and December 2019 for anonymous voluntary counseling and rapid testing (VCT) and/or medical consultations for sexually transmitted infections (STIs). The center provides anonymous and non-stigmatizing comprehensive sexual health services, including VCT and medical consultations, to all individuals regardless of their gender, sexual orientation, age, race, socio-economic background, or marital status. The data were stored in a secure electronic medical records (EMR) system.

Before data collection, the beneficiary was informed by the health professional that the latter will be administering an anonymous questionnaire prior to the testing and/or the medical consultation procedure and oral consent was obtained. The anonymous data from the questionnaire, rapid testing and medical consultations, were saved digitally under a file number that is unique for each beneficiary and can only be viewed by professionals working at the center and who have signed a confidentiality agreement. Beneficiaries were able to opt out from the questionnaire or from answering any specific question(s) without it affecting the delivery of services they requested. The research and all ethical considerations were approved by the IRB office of our institution (CE Université Saint-Joseph de Beyrouth #2016–77); research was performed in accordance with the good clinical practices described in the Helsinki declaration (version October 2013); informed consent was obtained from all participants or parents/legally authorized representatives of subjects that are under 18.

The inclusion criterion was being an unmarried cisgender woman (female born person who identifies as a woman). Only one visit was accounted for all beneficiaries. For those who visited the center more than once, the data from the first visit or the visit in which they tested positive for, or had a symptomatic diagnosis of an STI, were included.

### Data collection

The instrument, used previously^13^, included questions on demographics, sexual practices (including condom-use and condom-use discussion), and substance-use. Among demographic questions, the beneficiaries were asked about their sources of sexual health education (reliable sources were school, university and/or healthcare professional; unreliable sources were peers, sexual partners, and/or internet/pornography).

To assess condom-use, beneficiaries were asked about their condom-use behavior in the past three months with different types of partners: exclusive partners, regular partners (frequent but not exclusive), and one-time partners. Condom-use discussion with one-time partners was used to report whether the beneficiaries consistently discussed condom-use with their one-time partner(s) in the past three months. To assess prevalence of unwanted pregnancies, the women were asked if they have ever had an unwanted pregnancy. Unwanted pregnancy here is defined as a pregnancy that is neither planned nor wanted by the woman herself.

Substance-use was reported through questions on smoking, and recreational drug-use^13^. The term recreational drug-use in this study is defined as any use of a psychoactive drug to induce an altered state of consciousness for recreation in the past without a specific time frame.

Beneficiaries who underwent VCT had the option of being tested for HIV, Hepatitis B, Hepatitis C, and/or Syphilis using rapid tests (Abon). The results of the rapid tests were collected. Positive results were complemented by laboratory tests for confirmation (ELISA for HIV, VDRL for Syphilis, PCR for Hepatitis B and C) followed by a referral to an Infectious Diseases physician. The rapid test for Syphilis and Hepatitis C identifies the presence of treponema and Hepatitis C antibodies respectively, the rapid test for Hepatitis B detects the presence of the Hepatitis B surface antigen, while the HIV rapid test detects both the HIV surface antigen and HIV antibodies. As such, the rapid test for Syphilis and Hepatitis C do not necessarily indicate active infection.

Symptoms indicative of other STIs were identified through medical consultations by medical practitioners. These STIs include Neisseria Gonorrhea and/or Chlamydia Trachomatis, Ureaplasma Urealyticum/Parvum, Mycoplasma Genitalium, and Trichomonaisis (diagnosed by excessive and/or foul-smelling discharge and/or dysuria), and Human Papilloma Virus (HPV, diagnosed by genital warts).

Symptoms indicative of reproductive tract infections such as Yeast Infections or Bacterial Vaginosis were screened using a combination of the pH test, whiff test, wet mount, and potassium hydroxide solution (KOH).

### Statistics

Prevalence of HIV, Hepatitis B, Hepatitis C, Syphilis, Yeast Infections, Bacterial Vaginosis and symptoms indicative of Neisseria Gonorrhea and/or Chlamydia Trachomatis, Ureaplasma Urealyticum/Parvum, Mycoplasma Genitalium, Trichomonaisis, and genital warts were reported.

Univariate analysis (simple binomial regression) followed by multivariate stepwise binomial regression were computed to assess possible determinants for condom-use discussion, unwanted pregnancies, and recreational drug-use. The independent variables in these models include: age, sexual preference, source of sexual health education, occupation, highest level of education, recreational drug-use, cigarette smoking, number of partners, method of birth control, and vaginal sex exposure.

Univariate analysis followed by stepwise multinomial regression was performed to assess possible determinants of condom-use with partners in the past three months. The independent variables in this model include: Age, sexual preference, source of sexual health education, occupation, highest level of education, recreational drug-use, cigarette smoking, number of partners, method of birth control, and vaginal sex exposure.

Only variables with significant crude Odds Ratios (OR) in the univariate analysis, as well as demographics, were included in the multivariate analysis. Adjusted Odd Ratios (Adj. OR) with their respective *p*-value and confidence interval (CI) were reported for each model. The level of significance was set at 0.05. Statistics were performed under Xlstat (version 19.02, Addinsoft, Paris, France) and SPSS (version 20, IBM, Armonk, NY, USA).

## Results

A total of 7233 individuals voluntarily accessed the sexual health center between January 1st, 2015 and December 1st, 2019 of whom 2083 were cisgender unmarried women. Among the 2083 women who comprise the study sample, 1355 underwent only VCT, 189 received only medical consultations, and 539 underwent both VCT and medical consultations. As such a total of 1894 underwent VCT and 728 received medical consultations.

### Population characteristics

The age of the sample ranged between 15 and 60 years old, with a median age of 27 years (Q1: 24, Q3: 31). Data on demographics, sexual practices, and substance-use are summarized in Table 1. The majority were employed (67%) with a university level education (97%), had inconsistent condom-use (81%), and 0 or 1 partner in the past 3 months (63%). Only 2 women reported being sex-workers. Recreational drug-use was reported by 33% of the total sample.

**Table 1:** Demographics, substance use, sexual practices and sexual exposures in 2083 women in Lebanon.

Indicator	Missing values	Variables	Reported	Percentage (based on those who answered)
**Demographics**				
Age	0	< 20 years	50	2%
		20–24 years	568	27%
		25–29 years	826	40%
		30–34 years	415	20%
		35–39 years	140	7%
		≥ 40 years	84	4%
Sexual preferance	0	Woman having sex with men	1791	86%
		Woman having sex with women	97	5%
		Woman having sex with men and women	195	9%
Highest level of education	0	University	2011	97%
		Technical School	15	1%
		Highschool	38	2%
		Primary school	19	1%
Sources of sexual health education	0	Reliable Sources	281	13%
		Combination of reliable and unreliable sources	399	19%
		Unreliable Sources	1403	67%
Occupation	0	Employed	1399	67%
		Student	553	27%
		Unemployed	131	6%
Substance use				
Cigarette smoking	0	Does not smoke	790	38%
		Smokes	1293	62%
Recreational drug-use	0	Does not use recreational drugs	1406	67%
		Uses recreational drugs	677	33%
**Sexual practices**				
Condom-use with partner(s) in last 3 months	0	Always Uses Condoms	389	19%
		Inconsistant condom-use with exclusive partner	373	18%
		Inconsistant condom-use with regular partner	319	15%
		Inconsistant condom-use with one-time partner	1002	48%
Reasons for not using a condom	900	Trusts Partner	523	44%
		Heat of the moment	176	15%
		Was under the influence of a substance	135	11%
		Cannot negotiate condoms with partner	76	6%
		Partner loses erection	25	2%
		Makes them uncomfortable	140	12%
		Has misinformation about condom-use	108	9%
Condom-use discussion with one-time partner(s) in the last 3 months	0	Always discusses condom-use	1333	64%
		Does not always discuss condom-use	750	36%
Number of sexual partners in last 3 months	0	0 to 1	1322	63%
		2 to 5	706	34%
		≥ 6	55	3%
**Birth control**				
Birth control method	0	Condoms	1097	53%
		Oral Contraceptive Pills (OCPs)	297	14%
		Intrautirine Device (IUD)	70	3%
		Natural Methods (Withdrawel and/or calender rythm)	163	8%
		None	456	22%
History of unwanted pregnancy	302	Has never had an unwanted pregnancy	1593	89%
		Has had an unwanted pregnancy	188	11%
**Sexual exposure in last 3 months**				
Vaginal sex exposure	0	Did not have condomless vaginal sex	425	20%
		Had condomless vaginal sex	1658	80%
Anal sex exposure	1311	Did not have condomless anal sex	580	75%
		Had condomless anal sex	192	25%

### Prevalence of STIs and other infections

The rate of prevalence of HIV, Hepatitis B, Hepatitis C, Syphilis, symptoms of HPV, Neisseria Gonorrhea and/or Chlamydia Trachomatis, Ureaplasma Urealyticum/Parvum, Mycoplasma Genitalium, Trichomonaisis, Yeast Infections, and Bacterial Vaginosis are displayed in Table 2. Genital warts (15%) and symptoms indicative of Neisseria Gonorrhea and/or Chlamydia Trachomatis (14%) were relatively prevalent.

**Table 2:** Prevalence of STIs and other infections in 2083 women in Lebanon.

Infections	Total tested/diagnosed based on symptoms	Total positive	Percentages
**Diagnosis through rapid test**			
HIV	1882	2	< 1%
Hepatitis B	1622	1	< 1%
Hepatitis C	918	0	0
Syphilis	1498	1	< 1%
**Diagnosis based on symptoms**			
Genital warts	728	107	15%
Symptoms indicative of Neisseria Gonorrhea and/or Chlamydia Trachomatis	728	105	14%
Symptoms indicative of Ureaplasma Urealyticum/Parvum	728	61	8%
Symptoms indicative of Trichomonais	728	2	< 1%
Symptoms indicative of Mycoplasma Genitalium	728	2	< 1%
**Diagnosis based on combination of the pH test, whiff test, wet mount, and potassium hydroxide solution**			
Yeast Infection	728	95	13%
Bacterial Vaginosis	728	50	7%

### Condom-use and other sexual practices

Inconsistent condom-use during vaginal sex in the past three months was reported by 81% of the sample and having 2 or more partners by 37% (Table 1).

Among the reasons for not using a condom, the most common was trusting their partner(s) (44%), being in the heat of the moment (15%) and being under the influence of a substance (11%). Among those who reported not using a condom due to being under the influence of a substance, those substances were: 82% alcohol, 8% combination of recreational drugs, 5% combination of alcohol and recreational drug, and 5% of a single drug including cannabis, heroin, or cocaine.

Among those who reported inconsistent condom-use with one-time partners, 84% were women having sex with men, 11.5% were women having sex with both men and women, and 4.5% were women having sex with women.

The multinomial regression model on condom-use in the past three months (Table 3) showed that those who reported having 0–1 partner in the past three months were at lesser odds of having inconsistent condom-use with one-time partners compared to those who reported having 6 or more partners (adj. OR: 0.4).

**Table 3:** Multinomial regression model for condom-use with one-time partner(s) in the past three months with crude and adjusted odds ratios (ORs).

Condom-use: inconsistent condom-use with one-time partners	Co-variable	Crude OR	*p*-value	Adjusted OR	Confidence Interval	*p*-value
Age	< 20 years	1.7	0.33			
	20–24 years	0.7	0.28			
	25–29 years	0.85	0.62			
	30–34 years	0.74	0.38			
	35–39 years	0.81	0.59			
	≥ 40 years	Ref				
Sexual preference	Woman having sex with men	2.3	** < 0.001**	**2.2**	**1.4–3.5**	**0.001**
	Woman having sex with men and women	2.4	**0.002**	**2**	**1–3.3**	**0.03**
	Woman having sex with women	Ref				
Source of sexual education	Reliable	Ref				
	Various	1.3	0.16			
	Unreliable	0.9	0.7			
Highest level of education	University	Ref				
	Highschool	0.78	0.59			
Occupation	Employed	Ref				
	Student	0.99	0.93			
	Unemployed	0.75	0.28			
Recreational drug-use	Does not use	Ref				
	Uses	**1.8**	**0.002**	–	–	–
Cigarette smoking	Does not smoke	**0.73**	**0.01**	–	–	–
	Smokes	Ref				
Number of sexual partners in last 3 months	0 to 1	**0.38**	**0.01**	**0.4**	**0.18–0.87**	**0.01**
	2 to 5	0.82	0.62	0.84		0.68
	≥ 6	Ref				

Significant results are in bold

Those who reported consistently discussing condom-use with their one-time partner(s) accounted for 67%.

Binomial regression model on condom-use discussion with one-time partners (Table 4) showed that: (1) Those who were unemployed were at higher odds of not consistently discussing condom-use with partners (adj OR: 1.7); (2) Those who reported using recreational drugs were at higher odds of not consistently discussing condom-use with partners (adj. OR: 1.4). (3) Those who reported having 2 to 5 partners and 6 or more partners in the past three months were at higher odds of not consistently discussing condom-use with partners (adj. OR: 3.7, and 4.4 respectively).

**Table 4:** Binomial regression model for condom-use discussion with one-time partners with crude and adjusted odds ratios (ORs).

Condom-use discussion	Co-variable	Crude OR	*p*-value	Adjusted OR	Confidence Interval	*p*-value
Age	< 20 years	0.80	0.57			
	20–24 years	1.03	0.89			
	25–29 years	1.03	0.89			
	30–34 years	0.82	0.44			
	35–39 years	0.76	0.34			
	≥ 40 years	Ref				
Sexual preference	Woman having sex with men	1.1	0.6			
	Woman having sex with men and women	**2**	**0.007**			
	Woman having sex with women	Ref				
Source of sexual education	Reliable	Ref				
	Various	1	0.93			
	Unreliable	0.83	0.17			
Highest level of education	University	Ref				
	Highschool	0.89	0.75			
Occupation	Employed	Ref				
	Student	1.1	0.43	1.1	–	0.4
	Unemployed	**1.74**	**0.002**	**1.73**	**1.2–2.5**	**0.005**
Recreational drug-use	Does not use	Ref				
	Uses	**1.6**	** < 0.001**	**1.4**	**1.1–1.6**	**0.009**
Cigarette smoking	Does not smoke	0.92	0.37			
	Smokes	Ref				
Number of sexual partners in last 3 months	0 to 1	Ref				
	2 to 5	**3.8**	** < 0.001**	**3.7**	**3–4.5**	** < 0.001**
	≥ 6	**4.6**	** < 0.001**	**4.4**	**2.5–7.7**	** < 0.001**

Significant results are in bold

Among those who reported having one-time partners in the past three months (n = 784), 41% (n = 320) stated that they consistently discuss condom-use with their partner(s). Surprisingly, 58% (n = 183) among them reported condomless vaginal sex in the past three months.

### Unwanted pregnancies

Among those who responded to the question regarding ever having an unwanted pregnancy (n = 1781), 11% stated that they have had at least one unwanted pregnancy (Table 1).

The binomial regression model on having had an unwanted pregnancy (Table 5) showed that: (1) Those who were < 40 years old were significantly less likely to report ever having an unwanted pregnancy compared to those who were 40 years and above (adj. OR between 0.1 and 0.37). (2) Those who reported recreational drug-use were at higher odds of reporting having had an unwanted pregnancy (adj. OR: 2). (3) Those who reported their birth control method being an intrauterine device (IUD) and natural birth control methods (withdrawal and/or calendar rhythm) were at higher odds of ever having an unwanted pregnancy compared to those who reported condoms being their birth control method (adj OR: 2.9 and 2.4, respectively).

**Table 5:** Binomial regression model for unwanted pregnancies with crude and adjusted odds ratios (ORs).

Unwanted pregnancies	Co-variable	Crude OR	*p*-value	Adjusted OR	Confidence interval	*p*-value
Age	< 20 years	**0.09**	**0.02**	**0.1**	**0.02–0.8**	**0.03**
	20–24 years	**0.18**	** < 0.001**	**0.17**	**0.1–0.36**	** < 0.001**
	25–29 years	**0.41**	**0.005**	**0.37**	**0.2–0.71**	**0.003**
	30–34 years	0.70	0.27	0.7	0.36–1.3	0.29
	35–39 years	1.15	0.69	1.1	0.52–2.3	0.79
	≥ 40 years	Ref				
Sexual preference	Woman having sex with men	2.4	0.08			
	Woman having sex with men and women	2.1	0.18			
	Woman having sex with women	Ref				
Source of sexual education	Reliable	Ref				
	Various	1.18	0.47			
	Unreliable	0.94	0.84			
Highest level of education	University	Ref				
	Highschool	1.52	0.38			
Occupation	Employed	Ref				
	Student	**0.42**	** < 0.001**	–	–	–
	Unemployed	1.2	0.52			
Recreational drug-use	Does not use	Ref				
	Uses	**1.72**	** < 0.001**	**2**	**1.3–2.5**	** < 0.001**
Number of sexual partners in last 3 months	0 to 1	0.9	0.82			
	2 to 5	1.2	0.74			
	≥ 6	Ref				
Condom-use with partner(s) in last 3 months	Always uses condoms	**0.61**	**0.04**	–	–	–
	Inconsistent condom-use with exclusive partner	0.79	0.28			
	Inconsistent condom-use with regular partner	1.26	0.25			
	Inconsistent condom-use with one-time partner	Ref				
Method of birth control	Condoms	Ref				
	Oral contraceptive pills	1.19	0.46	1.19		0.47
	Intrauterine device (IUD)	**2.7**	**0.002**	**2.9**	**1.5–5.1**	**0.001**
	Natural methods	**2.2**	**0.002**	**2.4**	**1.4–4**	**0.005**
	None	0.82	0.29	0.84		0.39

Significant results are in bold

### Substance-use

**Figure 1:**
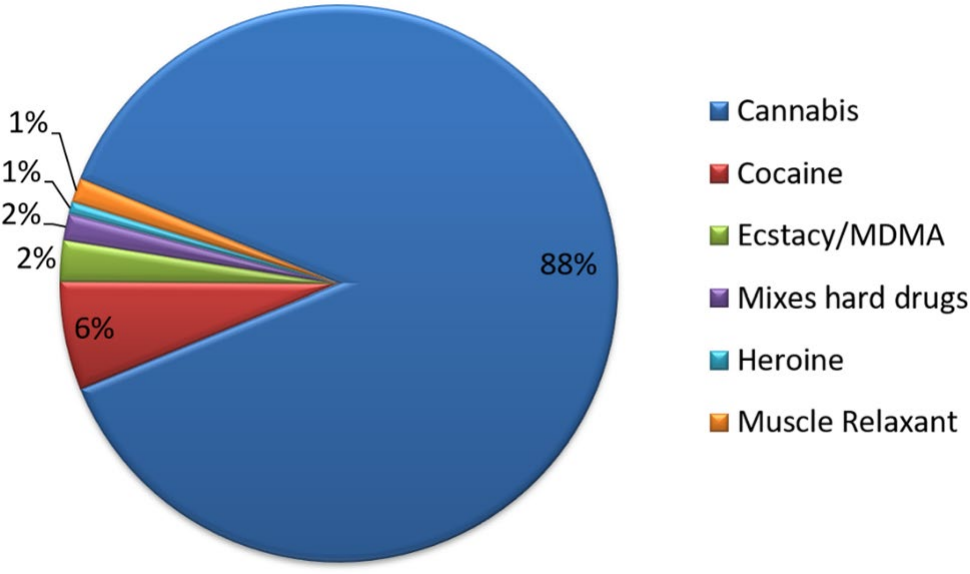
Recreational drugs used by 677 women.

Smoking cigarettes and/or water-pipe was reported among 63% of the sample, and recreational drug-use among 33%. The types of drugs used are displayed in Fig. 1. The recreational drug-use binomial regression model (Table 6) showed that: (1) Those who were less than < 40 years old were at greater odds of recreational drug-use compared to those who were 40 years and above (adj OR between 1.9 and 2.2). (2) Those who reported their sexual preference to be having sex with both men and women were at higher odds of recreational drug-use than those who reported their preference being having sex with women (adj. OR: 1.8). (3) Those who reported receiving sexual health education from a combination of reliable and unreliable sources were at higher odds of recreational drug-use than those who received sexual health education from reliable sources only (adj. OR: 1.9). (4) Those who did not smoke were at lesser odds of recreational drug-use (adj. OR: 0.6). (5) Those who reported consistently using condoms and those who had inconsistent condom-use with exclusive partners only were at lesser odds of recreational drug-use compared to those who had inconsistent condom-use with one-time partners (adj ORs: 0.72 and 0.7 respectively).

**Table 6:** Binomial regression model for recreational drug-use with crude and adjusted odds ratios (ORs).

Drug-use	Co-variable	Crude OR	p-value	Adjusted OR	Confidence Interval	*p*-value
Age	< 20 years	1.20	0.68	1.3	0.5–3.1	0.60
	20–24 years	**2**	**0.01**	**1.9**	**1.1–3.4**	**0.04**
	25–29 years	**2.2**	**0.004**	**2**	**1.2–3.7**	**0.02**
	30–34 years	**2.2**	**0.01**	**2.2**	**1.2–4**	**0.01**
	35–39 years	1.47	0.25	1.3	0.67–2.7	0.40
	≥ 40 years	Ref				
Sexual preference	Woman having sex with men	**0.62**	**0.03**	0.7		0.1
	Woman having sex with men and women	**1.7**	**0.02**	**1.8**	**1.1–3**	**0.01**
	Woman having sex with women					
Source of sexual education	Reliable	Ref				
	Various	**1.9**	** < 0.001**	**1.9**	**1.4–2.7**	** < 0.001**
	Unreliable	1.3	0.08	1.3	0.9–1.7	0.06
Highest level of education	University	Ref				
	Highschool	0.6	0.6			
Occupation	Employed	Ref				
	Student	0.88	0.24			
	Unemployed	1.1	0.63			
Smoking	Does not smoke	**0.6**	** < 0.001**	**0.6**	**0.5–0.75**	** < 0.001**
	Smokes	Ref				
Number of sexual partners in last 3 months	0 to 1	0.58	0.05			
	2 to 5	1.24	0.44			
	≥ 6	Ref				
Condom-use with partner(s)	Always uses condoms	**0.75**	**0.02**	**0.72**	**0.5–0.9**	**0.02**
	Inconsistent condom-use with exclusive partner	**0.62**	**0.01**	**0.70**	**0.5–0.8**	**0.02**
	Inconsistent condom-use with regular partner	0.89	0.39	0.84		0.22
	Inconsistent condom-use with one-time partner	Ref				
Vaginal sex exposure in the last 3 months	Did not have condomless vaginal sex	Ref				
	Had condomless vaginal sex	1.1	0.95			

Significant results are in bold

## Discussion

Women remain among those most vulnerable to the stigmatization of sex and sexuality, which subsequently hinders their ability to maintain their sexual health and well-being. In Lebanon, little is known about women’s sexual practices and their vulnerability to STIs. This deters our ability to provide evidence-based recommendations that will promote women’s sexual health and well-being. The lack of appropriate recommendations is particularly critical given the global increase in STIs and evidence of persistent barriers to SRH for women.

This is the first study in Lebanon and the MENA region to explore the prevalence of STIs and sexual health practices of cisgender unmarried women among a large sample (n = 2083). The majority reported condomless vaginal sex exposure in the past three months (80%), genital warts suggestive of HPV (15%) and symptoms indicative of gonorrhea/chlamydia (14%) were relatively prevalent. Recreational drug-use was also relatively prevalent (33%). Thus, the results of this study are essential for evidence-based recommendations to enhance access to SRH for women in Lebanon and similar contexts.

### STI prevalence

Rates of HIV, Hepatitis and Syphilis were low; however, symptoms associated with a bacterial/parasitic STI (Neisseria Gonorrhea and/or Chlamydia Trachomatis, Ureaplasma Urealyticum/Parvum, Mycoplasma Genitalium, and Trichomonaisis) were relatively high among the subset that underwent medical consultations. In fact, symptoms indicative of gonorrhea/chlamydia were higher than the prevalence reported in a local study on a sample of 505 women^14^. It is important to note that the reported rates are most likely underreported since these infections can often be asymptomatic. The high rates come in line with the global increase in STIs and reinforce the need for comprehensive SRH in Lebanon and the up-scaling of national surveillance for STIs.

The prevalence of genital warts of 15% was also higher than the rate reported in the local study^14^. Even though this study reported prevalence of HPV based on the presence of genital warts (caused by non-cancerous strains of HPV) evidence attests to an increased risk of HPV-related cancer among those who have had genital warts^15,16^.

Cervical cancer, the 6th most common cancer type among women aged 40 and less in Lebanon, is preventable through timely HPV vaccination^12^.However local sources attest to low level of HPV vaccination among women in Lebanon due to factors associated with the high cost of the vaccine as well as the lack of a uniform protocol for HPV vaccine recommendations for healthcare providers, allowing physicians’ personal biases to affect recommendations^17,18^.

Even though the high cost is the greatest barrier, social stigma also plays a role in decreasing demand for HPV vaccine especially among women who are not financially independent. Additionally, the limited access to non-stigmatizing information on sexual and reproductive health, especially in Arabic, may have also contributed to a decreased demand for the vaccine.

### Condom-use and discussion

The majority of the sample reported inconsistent condom-use with partners (81%) and having condomless vaginal sex in the past three months (80%) (Table 1). In congruence with these findings, one local source found that only 2 out of 63 female university students in Lebanon who had engaged in vaginal sex had ever used condoms, this was lower than the condom-use rate reported by their male counterparts^19^. Similarly in the region, condom-use was relatively low among a sample of Iranian women (26%) and was significantly lower than the rate among men from the same sample^20^.

The current study showed that women who had six or more partners in the past three months were found to be significantly more likely to have had inconsistent condom-use with one-time partners (Table 3). Having multiple partners was also found to be a significant predictor of condom-use among a sample of South African students^21^. Previous literature indicates a positive association between discussing condom-use with partners and using condoms^22^. Consistently discussing condom-use with partners (reported by 67% of the current study’s sample) was significantly less likely among women who used recreational drugs, were unemployed, or had multiple partners in the past three months (Table 4). It is likely that the lack of financial independence (unemployment) may have attributed to lower self-efficacy that could hinder condom-use discussion with partners. Additionally, recreational drug-users and women who reported having multiple partners may also have limited capacity to discuss condoms due to societal sigma associated with drug-use and sexual activity particularly against women.

However, the results also indicate that among the women who reported consistently discussing condom-use with one-time partners, more than half also reported having condomless vaginal sex in the past 3 months. Therefore, condom discussion, as reported by women in this sample, may not always be effective in increasing condom-use. This may be a result of the stigma surrounding women’s sexuality that is reinforced by the patriarchal norms in Lebanese society, which hinders women’s ability to discuss and demand condom-use with their partners. In fact women in conservative contexts may refrain from carrying condoms themselves because it is stigmatizing^4^. As such, women require more support to enhance their capacity to discuss and demand condom-use with their sexual partners.

### Birth control and unwanted pregnancies

The rate of women who reported using a reliable method of birth control (70%), such as condoms, oral contraceptive pills, and intra uterine devices, was higher than the rate reported in a local study on a sample of 825 married individuals in Lebanon (52%)^23^. Similarly, it was higher than the global rate of reliable birth control use reported by the United Nations in 2019 which was 44%^24^. The higher rates reported in this study are expected given that the women in the sample are sexually active, unmarried, and are actively seeking sexual health services at the center.

In 2012, the global rate of unintended pregnancies was at 40% of all pregnancies^25^. In Lebanon unwanted and/or unintended pregnancies (11% among this sample) increase women’s vulnerability to exploitation and unsafe abortions due to the illegality of abortion, with the exception of it being a threat to the mother’s life. Based on reported main method of birth control, 30% of the sample are not protected from unwanted pregnancies given that 8% reported using natural methods (such as withdrawal and/or calendar rhythm) and 22% having no method of birth control at all (Table 1).

Older women were significantly more likely to have had an unwanted pregnancy; this could be due to having a longer period of sexual activity than the younger women. Women who use recreational drugs were also more likely to have had an unwanted pregnancy. The positive association between substance-use and unwanted pregnancies was also established in a study of a sample of over 2000 women from the US^26^. In fact, the study finds that cannabis-users were also significantly more likely to have had an unwanted pregnancy, which corresponds to the recreational drug-users in this sample most of whom (88%) reported cannabis being their drug of choice. It is possible that recreational drug-users may engage in sex under the influence limiting their ability to use condoms. A study from Iran found that women substance-users can potentially internalize the stigma associated with substance-use and become more likely to engage in risky practices. This increases their vulnerability to unwanted pregnancies^8^.

Women who reported using natural methods of birth control and those who reported using intrauterine devices (IUDs) were more likely to have had an unwanted pregnancy compared to those who used condoms (Table 5). Given that this cross-sectional study is not temporal, the positive association between unwanted pregnancies and IUDs may be due to the women using IUDs after having had unwanted pregnancies as a means to avoid future unwanted pregnancies.

### Substance-use

The high prevalence of smoking (63%) among this sample was higher than the rate of cigarette smoking reported for Lebanon by the WHO in 2015 of 29%^27^. This difference is due to the smoking rate reported in this study incorporating both cigarette and water-pipe smoking, this latter being a popular trend among adults in Lebanon^28^.

There are no official estimates for the national prevalence of recreational drug-use in Lebanon, however some studies have found that substance-use has been increasing among youth and that the most common illicit substance used was cannabis^29^.

The rate of recreational drug-use (33%) was slightly higher than the rate reported among a sample of MSM residing in Lebanon (29%)^13^.

With the high rate of recreational drug-use an investigation into the possible associations was warranted. Sexually active women between the age of 20 and 39 and smokers were significantly more likely to use recreational drugs than older women and non-smokers.

Previous literature also found a significant association between cannabis-use and inconsistent condom-use^30,31^—This finding reiterates that recreational drug-use among women may lead to internalized stigma and negative self-perception^8^. These women may perceive that they are not entitled to demand safer practices, such as condom-use, from their partner(s).

This study has some limitations. First, the sample consists of sexually active unmarried cisgender women who voluntarily accessed the sexual health center and as such the findings cannot be generalized to the entire population. Given the sensitive nature of the questions and the stigma associated with sexual activity, reporting bias is possible. However, considering the entire process is anonymous and that the center is well-known for being a safe space, the potential for this bias is low. Another limitation is that most cases of diagnosis of HPV, and bacterial/parasitic STIs (other than Syphilis) were symptomatic and only few were confirmed through laboratory testing. This was necessary given the high cost of laboratory screening that many could not afford. The physicians providing the medical consultations are trained in the diagnosis and treatment of STIs and have ample experience; therefore, the potential for false diagnosis is low. However, given that gonorrhea, chlamydia, and HPV can be asymptomatic, the reported prevalence are likely underestimated. Moreover, the women in this study were not asked about whether they chose to end the unwanted pregnancy because even discussing the topic of abortion in Lebanon has a legal liability on the woman herself and the healthcare provider.

## Conclusion

Based on the findings of this study, the authors put forward the following recommendations:

1. The relatively high prevalence of genital warts and Gonorrhea/Chlamydia require a prompt national response to improve access to sexual health services including affordable prevention, screening and treatment services. Governmental agencies including the Ministry of Public Health, pharmaceutical companies, and health insurance companies should be lobbied to make the HPV vaccine available to all individuals in Lebanon for free or for a much more affordable fee. Furthermore, to improve screening for STIs nationally, it is essential that healthcare providers be trained on delivering non-stigmatizing sexual health services without discrimination based on race, age, drug-use, marital, social, or economic status.
2. The high level of inconstant condom-use reiterates the importance of providing comprehensive and non-stigmatizing sexual health education to all individuals in order to promote safe sexual practices and effective decision making with regards to consent, contraception, and condom-use. It is recommended to develop and disseminate accessible educational campaigns on various available methods of birth-control including information on how to access these methods via primary healthcare centers. It is also important to address the criminalization of abortion in Lebanon which eliminates women’s ability to make choices regarding their own bodies and exposes them to various dangers including exploitation and unsafe abortions.
3. A nationwide campaign on condom-use and negotiation is highly advised to support and improve women’s agency and capacity to discuss and demand condom-use with their partner(s) especially women who are not financially independent and those who use drugs. It is important to propagate the understanding that condom-use is equally the responsibility of women and their partner(s) and should not only be initiated by men as is common in conservative cultures^4^.
4. The high rates of recreational drug-use and associated inconsistent condom-use show a need for accessible harm reduction services including information on safe sexual practices for people engaging in sex under the influence.
5. Finally, providing accessible, non-stigmatizing, and inclusive sexual health information and services will contribute to breaking the taboo surrounding women’s sexuality in the Lebanese context and alleviate associated stigma.
